# Insights Into Comparative Analyses and Phylogenomic Implications of *Acer* (Sapindaceae) Inferred From Complete Chloroplast Genomes

**DOI:** 10.3389/fgene.2021.791628

**Published:** 2022-01-03

**Authors:** Tao Yu, Jian Gao, Pei-Chun Liao, Jun-Qing Li, Wen-Bao Ma

**Affiliations:** ^1^ CECEP Eco-Product Development Research Center, Beijing, China; ^2^ Forestry College, Beijing Forestry University, Beijing, China; ^3^ Faculty of Resources and Environment, Baotou Teachers’ College, Inner Mongolia University of Science and Technology, Baotou, China; ^4^ Department of Life Science, National Taiwan Normal University, Taipei, Taiwan; ^5^ Key Laboratory of National Forestry and Grassland Administration on Sichuan Forest Ecology and Resources and Environment, Sichuan Academy of Forestry, Chengdu, China

**Keywords:** *Acer*, chloroplast genome, sequence divergence, structural variation, phylogenetics

## Abstract

*Acer* L. (Sapindaceae) is one of the most diverse and widespread plant genera in the Northern Hemisphere. It comprises 124–156 recognized species, with approximately half being native to Asia. Owing to its numerous morphological features and hybridization, this genus is taxonomically and phylogenetically ranked as one of the most challenging plant taxa. Here, we report the complete chloroplast genome sequences of five *Acer* species and compare them with those of 43 published *Acer* species. The chloroplast genomes were 149,103–158,458 bp in length. We conducted a sliding window analysis to find three relatively highly variable regions (*psbN*-*rps14*, *rpl32*-*trnL*, and *ycf1*) with a high potential for developing practical genetic markers. A total of 76–103 SSR loci were identified in 48 *Acer* species. The positive selection analysis of *Acer* species chloroplast genes showed that two genes (*psaI* and *psbK*) were positively selected, implying that light level is a selection pressure for *Acer* species. Using Bayes empirical Bayes methods, we also identified that 20 cp gene sites have undergone positive selection, which might result from adaptation to specific ecological niches. In phylogenetic analysis, we have reconfirmed that *Acer pictum* subsp. *mono* and *A*. *truncatum* as sister species. Our results strongly support the sister relationships between sections *Platanoidea* and *Macrantha* and between sections *Trifoliata* and *Pentaphylla*. Moreover, series *Glabra* and *Arguta* are proposed to promote to the section level. The chloroplast genomic resources provided in this study assist taxonomic and phylogenomic resolution within *Acer* and the Sapindaceae family.

## 1 Introduction

With the rapid development of next-generation sequencing (NGS), the increasing chloroplast (cp) genome sequences of land plants offer comprehensive comparison in genome structure, horticultural improvement in plant breeding (Sonah et al., 2011; Xiong et al., 2015), and phylogenetic reconstruction (Cai et al., 2015; Ruhsam et al., 2015). The cp genome is maternally inherited with high copy numbers per cell, despite being much smaller than other genomes (Yi et al., 2013). The cp genome is commonly used in evolution and phylogenomic analysis, providing supplementary information hidden in nuclear genomes regarding, for instance, ancient taxa histories and population-area relationships (Timme et al., 2007; Zeb et al., 2019). The cp genome’s relatively conserved features make it being broadly applied to plant systematics, biodiversity, biogeography, adaptation, etc. (Wambugu et al., 2015; Brozynska et al., 2016).

*Acer* L. (Maple), composed of more than 124 species, is a diverse genus within the Sapindaceae L. family (Xu et al., 2008), which are primarily deciduous and distributed in temperate Asia, Europe, and North America (van Gelderen et al., 1994; Renner et al., 2008; Xu et al., 2008). Many *Acer* species provide important economic products, such as timber, furniture, and herbal medicines, especially gamma-linolenic acid, and the genus also includes many famous horticultural plants (Bi et al., 2016). Moreover, some *Acer* species are dominant in several forests, responsible for fundamental ecosystem processes (Bishop et al., 2015). High variable leaf characters and complex reproductive characteristics hinder *Acer*’s systematic classification (Cronquist, 1979; Rosado et al., 2018). An accurate phylogeny can facilitate the sustainable utilization of wild genetic resources (Xu et al., 2008). Previously, the phylogenetic trees of *Acer* have been reconstructed by cambial peroxidase isozymes (Santamour, 1982), restriction fragment length polymorphism (RFLP) markers (Pfosser et al., 2002), cp DNA and nuclear DNA (Cho et al., 1996; Ackerly and Donoghue, 1998; Li et al., 2006; Renner et al., 2008; Li et al., 2019; Gao et al., 2020), and cp genome (Areces-Berazain et al., 2020; Wang et al., 2020; Yu et al., 2020; Areces-Berazain et al., 2021). However, limited informative sites, taxa, and evolution models used for the phylogenetic analyses led to the phylogenetic relationship being poorly resolved. Therefore, large-scale plastome data is necessary to acquire a maximum phylogenetic signal in *Acer*.

In this study, we compiled a dataset with the cp genomes of 48 *Acer* species, five of which were newly generated in this study (*A. palmatum*, *A. wilsonii*, *A. flabellatum*, *A. sino-oblongum*, and *A. laevigatum*). Because of the importance of plastomes in systematics, it is necessary to confirm these plastomes’ gene order and sequence homology. Therefore, by comparing plastome studies, we aimed: 1) to determine the gene order and gene content of *Acer* cp genomes, 2) to identify divergence hotspots and the positive selective genes in the cp genomes, and 3) to reconstruct the phylogenomic relationships of *Acer* species.

## 2 Materials and Methods

### 2.1 Sampling and DNA Extraction

Young leaves of five *Acer* species (*A. palmatum*, *A. wilsonii*, *A. flabellatum*, *A. sino-oblongum*, and *A. laevigatum*) were collected and dried immediately with silica gel for DNA extraction with the modified CTAB method (Doyle, 1987). The sampling information is shown in Supplementary Table S1. Species identification was followed by *Maples of the World* (van Gelderen et al., 1994) and *Flora of China* (Xu et al., 2008). Voucher specimens were deposited at the College of Forestry, Beijing Forestry University, China.

### 2.2 Chloroplast Genome Sequencing, Assembling, and Annotation

Purified genomic DNA was sequenced using an Illumina MiSeq sequencer (Shanghai OE Biotech Co., Ltd.). A paired-end library was constructed with an insert size of 300 bp, yielding at least 8 GB of 150 bp paired-end reads for each species. Clean reads were obtained with NGSQC Toolkit v2.3.3 (cut-off read length for HQ = 70%, cut-off quality score = 20, trim reads from 5′ = 3, trim reads from 3′ = 7) (Dai et al., 2010). MITObim v. 1.8 (Hahn et al., 2013) was used to assemble the following reference cp genomes: *A*. *buergerianum* subsp. *ningpoense* (KF753631) (Yang et al., 2015), *A*. *miaotaiense* (KX098452) (Zhang et al., 2016), *A*. *davidii* (KU977442) (Jia et al., 2016), and *A*. *morrisonense* (KT970611) (Li et al., 2017). Annotation was performed using DOGMA (Wyman et al., 2004). Protein-encoding genes (PCG), tRNAs, rRNAs were annotated by BLAST searches (https://blast.ncbi.nlm.nih.gov/Blast.cgi) with manual adjustment error. The boundaries between the representative *Acer* cp genome regions were determined with the online tool IRscope (Amiryousefi et al., 2018), and ten representative species form main groups of *Acer* were highlighted.

### 2.3 Identifying Cp SSRs

MISA (MIcroSAtellite, http://pgrc.ipk-gatersleben.de/misa/) was used to detect simple sequence repeats (SSRs) with criteria of minimal repeat numbers ten in mono-nucleotide SSR, four in di- and tri-nucleotide, and three in tetra-, penta-, and hexa-nucleotide SSRs motifs.

### 2.4 Divergence Hotspot Identification

Cp sequences were aligned by MAFFT (Katoh et al., 2005), and sliding window analysis was then used to estimate nucleotide variation (π) with 600-bp window length and 200-bp step size using DnaSP 5.0 (Librado and Rozas, 2009).

### 2.5 Positive Selected Analysis

The CodeML program in PAML 4.7.1 (Yang, 2007) was used to test the positive selection of *Acer* cp genes under the site-specific models. The dN, dS, and ω (= dN/dS) values were calculated with seqtype = 1, model = 0, Nssites = 0, 1, 2, 7, 8 based on 77 protein-coding genes shared by 48 *Acer* species. A maximum-likelihood phylogenetic tree was reconstructed using whole cp genomes by PhyML v3.0 (Guindon et al., 2005). Likelihood ratio tests (LRT) were used to compare models between M1 (neutral) and M2 (positive selection) and between M7 (beta) and M8 (beta and ω). *p*-value was calculated using the internal CHI2 program in PAML 4.7.1 (Yang, 2007).

### 2.6 Phylogenomic Reconstruction

To reconstruct the phylogeny, 58 cp genome sequences comprising five new plastome sequences, 43 plastomes of *Acer* species from GenBank, and ten outgroup species were used (Supplementary Table S2). BioEdit version 7.1.11 (Hall, 1999) was used to align sequences with manual refinement and finally generated a total of 184,290 bp alignment length. The 5ʹ and 3ʹ ends of the sequences were trimmed to equal lengths for subsequent phylogenetic analyses. Phylogenetic relationships were reconstructed using Bayesian inference (BI), maximum likelihood (ML), and maximum parsimony (MP) by MrBayes 3.2 (Ronquist et al., 2012), PhyML v3.0 (Guindon et al., 2005), and PAUP*4.0b10 (Swofford, 2003), respectively. The best-fitting substitution model (GTR + I + G) was determined using Modeltest 3.7 (Posada and Buckley, 2004). In the Bayesian analyses, two independent Markov Chain Monte Carlo (MCMC) permutations were initiated. Each consisted of one cold and three heated MCMC chains for 10^8^ generations and sampled every 10^4^ generations. The first 2,000 trees were discarded as burn-in to ensure that the chains had become stationary. The ML analysis was initiated from a BIONJ tree, with support values for the nodes estimated by 1,000 bootstrap replicates. In the MP analysis, all character states were treated as unordered and equally weighted, and a heuristic search was performed with 1,000 replicates of random addition of sequences, tree-bisection-reconnection branch-swapping, and MULTREES. Bootstrap analysis was conducted in 1,000 replicates with the same heuristic search settings described above.

## 3 Results and Discussion

### 3.1 Choroplast Genome Organization of *Acer*

The nucleotide sequences of the 48 *Acer* cp genomes ranged from 149,103 bp (*A. paxii*) to 158,458 bp (*A*. *caudatifolium*) (Table 1). These cp genomes revealed a typical quadripartite structure similar to most angiosperms, with LSC, SSC, and IRs (IRa and IRb) regions. The LSC, SSC, and IR regions were 78,768–86,911 bp, 17,474–18,232 bp, and 25,508–26,798 bp long, respectively (Table 1). The guanine (G) and cytosine (C) proportion (GC%) varied from 37.5 to 38.1%, in which 34 species have a stable GC content of 37.9%. The GC content was higher in the IR region than the LSC and SSC regions.

**TABLE 1:** General features of the *Acer* chloroplast genomes compared in this study.

Species	Total (bp)	GC (%)	LSC (bp)	SSC (bp)	IR (bp)	Accession no
*Acer acuminatum*	155,548	37.9	85,358	18,046	26,072	MN864496
*Acer amplum*	156,225	37.9	86,121	18,066	26,019	NC034932
*Acer buergerianum* subsp*. ningpoense*	156,911	37.9	85,315	18,094	26,751	NC034744
*Acer caesium* subsp. *giraldii*	154,176	38.1	82,759	17,895	26,761	MK479225
*Acer cappadocicum*	157,353	37.9	85,723	18,040	26,798	NC051956
*Acer carpinifolium*	155,212	38.0	85,448	17,724	26,020	MN864497
*Acer catalpifolium*	157,349	37.9	85,745	18,066	26,769	MF179637
*Acer caudatifolium*	158,458	37.8	86,911	18,059	26,744	MK479226
*Acer cinnamomifolium*	156,227	37.9	85,928	18,121	26,079	NC056164
*Acer cissifolium*	155,997	37.9	85,790	18,051	26,078	MW067037
*Acer davidii*	157,044	37.9	85,410	18,112	26,761	KU977442
*Acer fenzelianum*	156,535	37.9	85,166	18,077	26,646	NC045527
*Acer flabellatum*	156,472	37.9	84,876	18,088	26,754	MF787384^a^
*Acer tataricum* subsp. *ginnala*	156,184	38.1	85,485	18,032	26,047	MN864511
*Acer glabrum*	156,373	37.9	86,034	18,211	26,064	MN864498
*Acer griseum*	156,857	37.9	85,227	18,134	26,748	KY511609
*Acer henryi*	156,325	37.9	86,034	18,097	26,097	MW067048
*Acer laevigatum*	156,905	37.9	85,323	18,084	26,749	MF521832^a^
*Acer longipes*	157,137	37.9	85,531	18,068	26,769	MG751775
*Acer lucidum*	157,612	38.1	86,838	18,094	26,340	MK479214
*Acer mandshuricum*	156,234	37.9	86,043	18,059	26,066	MW067055
*Acer miaotaiense*	156,595	37.9	86,327	18,068	26,100	KX098452
*Acer micranthum*	156,399	37.9	86,147	18,128	26,062	MN864500
*Acer morrisonense*	157,197	37.8	85,655	18,086	26,728	KT970611
*Acer negundo*	155,938	37.9	85,678	18,092	26,084	MN841452
*Acer nikoense*	156,082	37.9	85,866	18,148	26,034	MN864499
*Acer nipponicum*	156,225	37.8	85,823	18,232	26,085	MN864502
*Acer oblongum*	155,686	38.0	85,665	17,821	26,100	NC056208
*Acer palmatum*	157,023	37.9	85,342	18,167	26,757	KY457568^a^
*Acer paxii*	149,103	37.5	78,768	17,474	26,366	MK479215
*Acer pentaphyllum*	156,220	37.9	85,938	18,148	26,067	MN864505
*Acer pictum* subsp. *mono*	156,985	37.9	85,378	18,069	26,769	MG751776
*Acer pilosum*	155,586	38.0	85,313	18,139	26,076	MN864506
*Acer platanoides*	156,385	37.9	86,098	18,107	26,090	NC051959
*Acer pseudosieboldianum*	157,053	37.9	85,392	18,169	26,746	MW067066
*Acer robustum*	156,790	37.9	85,127	18,115	26,774	MK479212
*Acer rubrum*	155,683	37.9	85,383	18,086	26,107	MN864509
*Acer saccharum*	155,684	37.9	85,393	18,033	26,129	NC051960
*Acer sino*-*oblongum*	157,121	37.9	85,558	18,119	26,722	KY987160^a^
*Acer sterculiaceum* subsp. *sterculiaceum*	156,258	38.0	86,014	18,048	26,098	MN864510
*Acer sutchuenense* subsp. *tienchuanense*	156,063	37.9	85,127	18,115	26,774	NC049166
*Acer takesimense*	157,023	37.9	85,371	18,160	26,746	NC046488
*Acer tegmentosum*	156,435	37.8	86,139	18,103	26,097	NC056233
*Acer tetramerum*	154,078	38.1	83,199	17,895	26,492	MK479228
*Acer truncatum*	156,262	37.9	86,019	18,073	26,085	MH716034
*Acer wilsonii*	157,067	37.9	85,419	18,128	26,760	MG012225^a^
*Acer yangbiense*	155,706	38.0	86,593	18,097	25,508	MN315285
*Acer yangjuechi*	157,088	37.9	85,483	18,069	26,768	MG770234

a Sequences obtained in this study.

A total of 117 genes included four unique rRNAs, 31 tRNAs, and 82 PCGs (Table 2). Most cp genes were single copy, whereas 23 genes exhibited double copies, including four rRNA (*4.5S*, *5S*, *16S*, and *23S* rRNA), nine tRNA genes (*trnA-UGC*, *trnI-CAU*, *trnI-GAU*, *trnL-CAA*, *trnM-CAU*, *trnN-GUU*, *trnR-ACG*, *trnT-GGU*, and *trnV-GAC*), and 10 PCG (*ndhB*, *rpl2*, *rps12*, *rpl23*, *rps19*, *rps7*, *ycf1*, *orf42*, *ycf2*, and *ycf15*). A total of 18 genes had introns, and three genes (*ycf3*, *clpP,* and *rps12*) contained two introns. Despite typically highly conserved, gene relocation and structural variation in IR and single-copy regions are very common (de Santana Lopes et al., 2018; Shearman et al., 2020; Guo et al., 2021). The cp genome structures of 10 representative *Acer* species are shown in Supplementary Figure S1. Two main types of *Acer* species were recognized: the first group was represented by *A. catalpifolium*, *A. buergerianum*, *A. negundo*, whose LSC-IRB junction region comprised the *rpl22* gene; the second group was composed of *A*. *micranthum*, *A*. *lucidum*, *A*. *yangbiense*, *A*. *tataricum* subsp. *ginnala*, *A. carpinifolium*, *A*. *glabrum*, and *A*. *caesium*, whose LSC-IRB junction comprised the *rps19* or *rpl2* gene regions, or the spacer region between *rps19* and *rpl2*. The structure of the three species in the first group was relatively stable and had the same distance between *rpl22* and the LSC-IRB junction. However, in the second group, the distance of *rps19* and *rpl2* from the LSC-IRB junction significantly varied. These structural pattern variations are similar to those of Saxifragaceae species (Li et al., 2019). Compared with the LSC-IRB junction, the SSC-IRB junction showed clear conservativeness, except for the deletion of pseudogene *ycf1* (φ *ycf1*) in *A. trigonatum*. SSC-IRB junctions of *Acer* species were all located in the *ycf1* gene, and the length of the *ycf1* fragment in the IRB region was 1,244–1,284 bp. The length of the *ndhF* gene starting site from the SSC-IRB junction was 32–48 bp.

**TABLE 2:** Genes present in the *Acer* chloroplast genome.

Group of gene	Genes name
Photostsyem I	*psaA*, *psaB*, *psaC*, *psaI*, *psaJ*
Photostsyem II	*psbA*, *psbB*, *psbC*, *psbD*, *psbE*, *psbF*, *psbh*, *psbI*, *psbJ*, *psbK*, *psbL*, *psbM*, *psbN*, *psbT*, *psbZ*
Cytochrome b/f complex	petA, petB*, petD*, *petG*, *petL*, *petN*
ATP synthase	*atpA*, *atpB*, *atpE*, atpF*, *atpH*, *atpI*
NADH dehydrogenase	ndhA*, ndhB*, *ndhC*, *ndhD*, *ndhE*, *ndhF*, *ndhG*, *ndhH*, *ndhI*, *ndhJ*, *ndhK*
RubisCO large subunit	*rbcL*
RNA polymerase	*ropA*, *ropB*, ropC1*, *ropC2*
Ribosomal proteins (SSU)	*rps2*, *rps3*, *rps4*, *rps7*, *rps8*, *rps11*, rps12**, *rps14*, *rps15*, rps16*, *rps18*, *rps19*
Ribosomal proteins (LSU)	rpl2*, *rpl14*, rpl16*, *rpl20*, *rpl22*, *rpl23*, *rpl32*, *rpl33*, *rpl36*
Other gene	clpP**, *matK*, *accD*, *ccsA*, *infA*, *cemA*
Proteins of unknown function	*ycf1*, *ycf2*, ycf3**, *ycf4*, *ycf15*
ORFs	*Orf42*
Transfer RNAs	31 tRNAs (six contain a single intron)
Ribosomal RNAs	*rrn4.5*, *rrn5*, *rrn16*, *rrn23*

A single asterisk (*) preceding gene names indicate intron-containing genes, and double asterisks (**) preceding gene names indicate two introns in the gene.

### 3.2 SSRs Analysis of the *Acer* Cp Genomes

**FIGURE 1:**
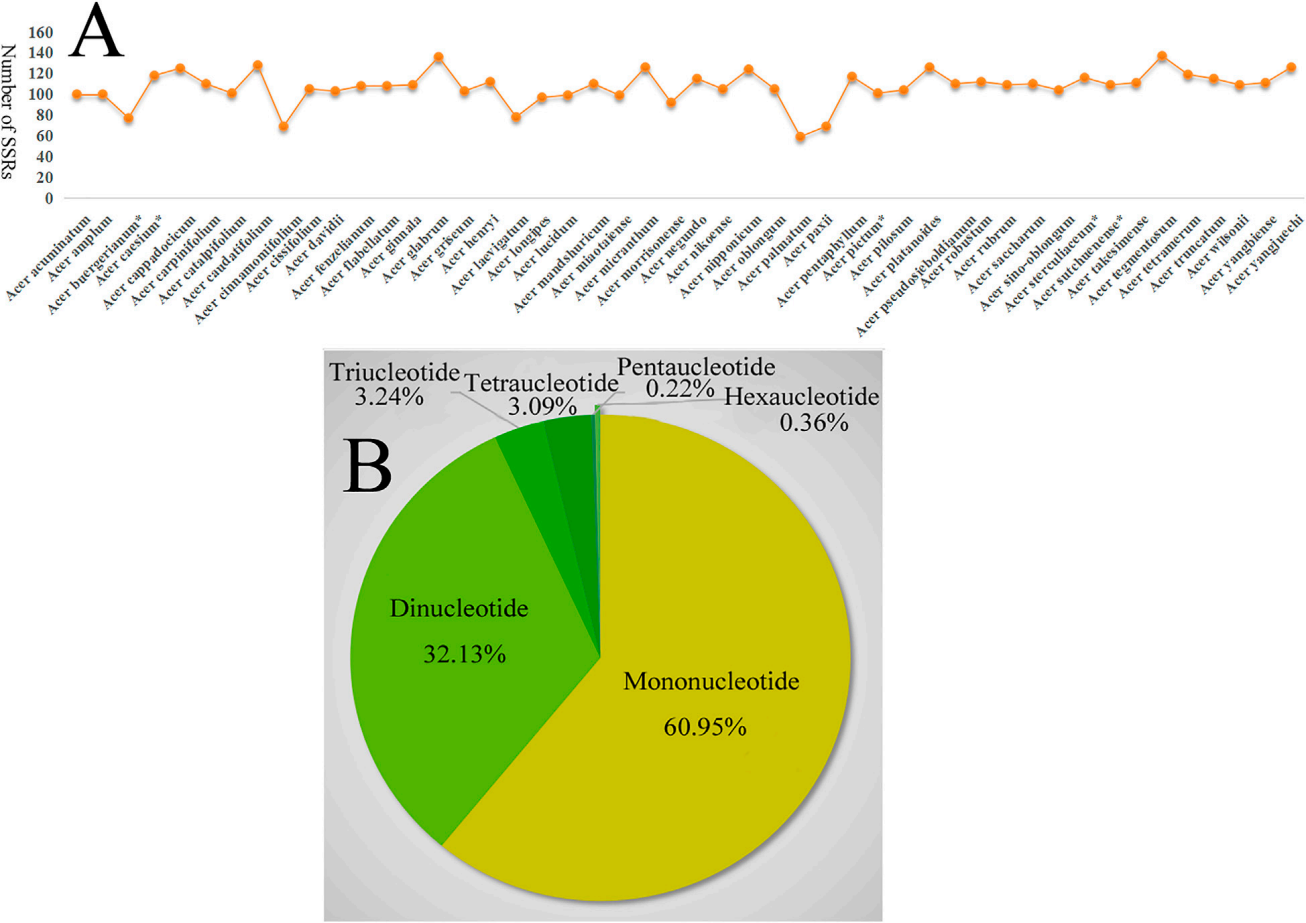
**(A)** Number of SSRs in the *Acer* species chloroplast genome. **(B)** Number of SSRS types of chloroplast genome in 48 *Acer* species.

A total of 5,136 SSR loci were detected in the 48 *Acer* species, with the highest number in *A. tegmentosum* (137) and the lowest number in *A. palmatum* (59) (Figure 1A), in which six SSR types in *A. negundo*, five types in 21 species, four types in other 24 species, and three types in the remaining two species (*A*. *palmatum* and *A*. *buergerianum*). Most SSRs were mono- and di-nucleotide motifs; the former was the most abundant SSR type, being detected at 3,251 loci (60.95% of the total number), while 1,714 di-nucleotide repeats (32.13%) were detected. The least frequent type was penta-nucleotide, which was detected in only 12 loci in all *Acer* species (Figure 1B). The mono-nucleotide SSRs mostly comprised short polyA and polyT repeats, which have also been reported in other species, including *Salvia miltiorrhiza* (Lamiaceae) (Qian et al., 2013) and three *Veroniceae* species (Plantaginaceae) (Choi et al., 2016). Most SSRs were detected in intergenic regions. Within the coding regions, the SSRs were concentrated in *ycf1* and *ycf2*, which is consistent with other species such as *Cynara cardunculus* (Curci et al., 2015) and *Vigna radiata* (Tangphatsornruang et al., 2009). Thus, the highly variable *ycf1* coding region may potentially be applied as an alternative marker for plastid candidate barcodes to solve the phylogenetic controversy (Dong et al., 2015). SSR information may be crucial for understanding the genetic diversity status of *Acer* species worldwide.

### 3.3 Divergence Hotspot of *Acer* Species

**FIGURE 2:**
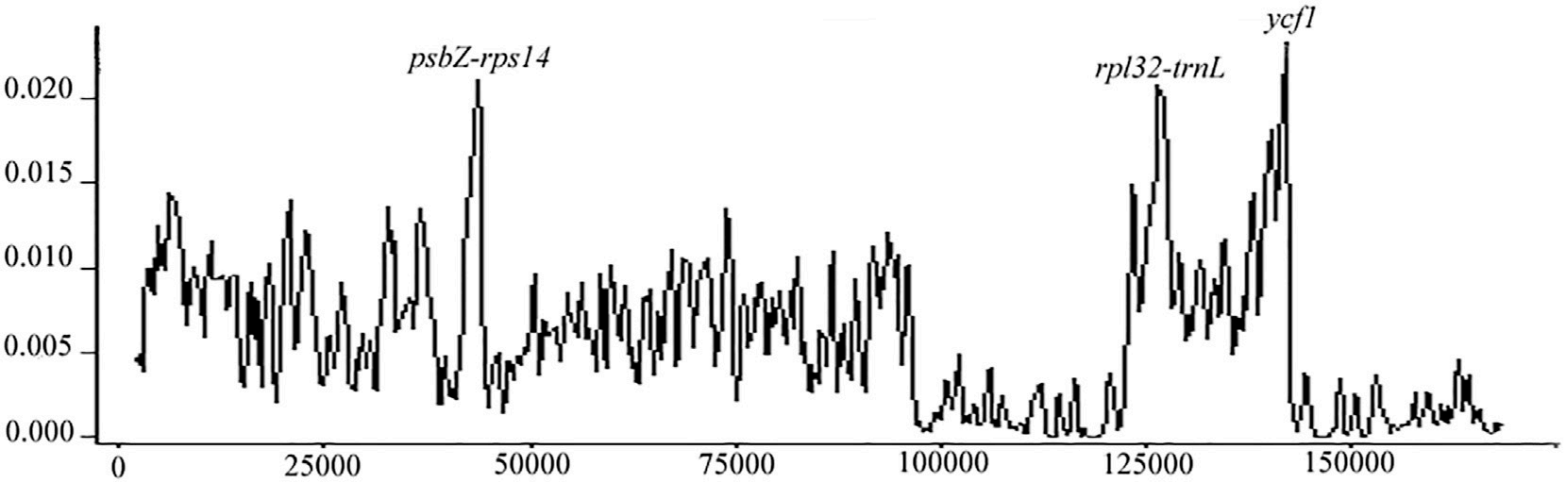
Sliding-window analysis on the cp genomes for *Acer* species.

The sliding window analysis showed that nucleotide variability was higher in *psbZ-rps14*, *rpl32-trnL*, and *ycf1* than in other regions (Figure 2). Maximum nucleotide polymorphism was 0.023, showing that those cp genomes were relatively conserved.

One highly variable region was found in the LSC region, and two were distributed in the SSC region, indicating the most stable region in the IR, followed by the LSC. In the *Acer* section *Platanoidea*, the *trnH*-*psbA*, *psbN*-trn*D*, *psaA*-*ycf3*, *petA*-*psbJ*, and *ndhA* introns were suggested as highly variable (Yu et al., 2020). With a comparison of 16 *Acer*, Areces-Berazain et al. (2020) defined the most variable regions in the SSC, in which *ycf1*, *ndhF*-*rpl32*, and *rpl32-trnL* had the highest nucleotide polymorphisms (Areces-Berazain et al., 2020). Accordingly, we concluded that the SSC region could apply for molecular barcoding in *Acer*, where *rpl32-trnL* and *ycf1* are the most appropriate candidates. The function of the *ycf1* gene in the cp genome has not been determined and is generally treated as an open reading frame (Dong et al., 2012). The *ycf1* gene, which showed high polymorphism in previous studies, may be designed as the molecular marker for phylogenetic analyses (Dong et al., 2015; He et al., 2017).

### 3.4 Positive Selection Analysis

**FIGURE 3:**
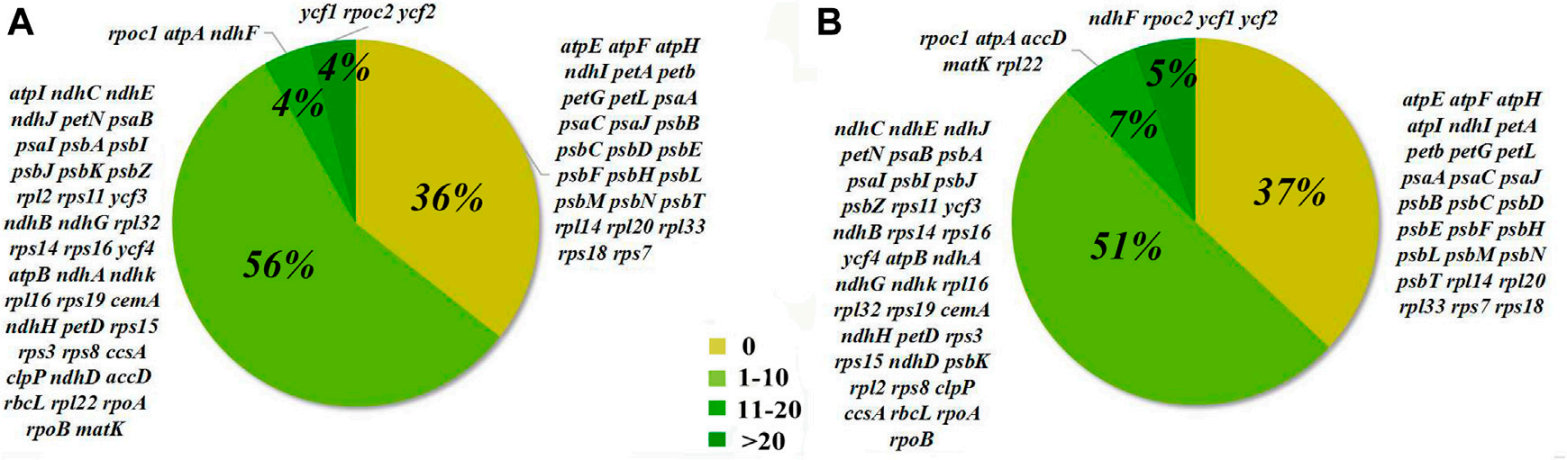
Percentages of the number of sites selected from 73 coding genes in 48 *Acer* species. A, analysis results of Model M2. B, analysis results of Model M8.

Seventy-three protein-coding gene sites were identified to be positively selected under the CodeML codon substitution models. Two genes (*psaI* and *psbK*) were detected to be positively selected with ω > 1 under the one-ratio model (M0), and nine genes (*rps8*, *rpoC2*, *rps16*, *ycf1*, *ndhG*, *matK*, *rpl22*, *petN*, and *ycf2*) with ω between 0.5 and 1.0, indicating relaxation of selective constraint. We also identified cp genes with sites under positive selection in models M2 and M8, which rejected the null models M1 and M7, respectively (Table 3). In model M2, 41 genes had 1–10 sites, three genes had 11–20 sites, and three genes had more than 20 sites under positive selection. In model M8, 37 genes had 1–10 sites, five genes had 11–20 sites, and four genes had more than 20 sites under positive selection (Figure 3). Among them, 20 genes have significantly positively-selected sites based on Bayes empirical Bayes (BEB) posterior probability, including two subunits of the ATP gene (*atpA* and *atpB*), three NADH dehydrogenase genes (*ndhA*, *ndhD*, and *ndhH*), one of the cytochrome b/f complex genes (*petD*), one of Photostsyem I (*psaI*), one of RubisCO large subunit gene (*rbcL*), four RNA polymerase genes (*ropA*, *ropB*, *ropC1,* and *ropC2*), three ribosomal protein genes (*rps8*, *rps11*, and *rps19*), and *accD*, *clpP*, *ycf1*, *ycf2*, and *ycf3*.

**TABLE 3:** Detection of positive selection sites of chloroplast genes in *Acer* genus.

Genes	Model	Parameters	2ΔL	Sites
*accD*	M0 (one ratio)	ω = 0.312		
	M1 (neutral)	−3,194.888	4.319	
	M2 (selection)	−3,192.729		8
	M7 (beta)	−3,194.917	4.405	
	M8 (beta&ω)	−3,192.714		13
*atpA*	M0 (one ratio)	ω = 0.414		
	M1 (neutral)	−3,062.544	68.257	
	M2 (selection)	−3,028.416		12
	M7 (beta)	−3,062.550	68.092	
	M8 (beta&ω)	−3,028.500		12
*atpB*	M0 (one ratio)	ω = 0.195		
	M1 (neutral)	−2,767.483	19.820	
	M2 (selection)	−2,757.573		3
	M7 (beta)	−2,768.353	21.511	
	M8 (beta&ω)	−2,757.597		3
*clpP*	M0 (one ratio)	ω = 0.290		
	M1 (neutral)	−1,222.189	75.140	
	M2 (selection)	−1,184.619		5
	M7 (beta)	−1,222.474	70.978	
	M8 (beta&ω)	−1,186.985		6
*ndhA*	M0 (one ratio)	ω = 0.215		
	M1 (neutral)	−2,101.206	16.580	
	M2 (selection)	−2092.916		3
	M7 (beta)	−2,101.626	17.610	
	M8 (beta&ω)	−2092.821		3
*ndhD*	M0 (one ratio)	ω = 0.238		
	M1 (neutral)	−2,926.687	24.031	
	M2 (selection)	−2,914.671		5
	M7 (beta)	−2,926.898	24.425	
	M8 (beta&ω)	−2,914.686		5
*ndhF*	M0 (one ratio)	ω = 0.398		
	M1 (neutral)	−5,574.424	97.807	
	M2 (selection)	−5,525.520		13
	M7 (beta)	−5,577.921	109.337	
	M8 (beta&ω)	−5,523.252		22
*PetD*	M0 (one ratio)	ω = 0.271		
	M1 (neutral)	−942.151	11.366	
	M2 (selection)	−936.469		4
	M7 (beta)	−942.381	11.793	
	M8 (beta&ω)	−936.484		4
*psaI*	M0 (one ratio)	ω = 3.320		
	M1 (neutral)	−190.292	23.988	
	M2 (selection)	−178.298		2
	M7 (beta)	−192.000	27.404	
	M8 (beta&ω)	−178.298		2
*rbcL*	M0 (one ratio)	ω = 0.323		
	M1 (neutral)	−2,649.421	94.692	
	M2 (selection)	−2,602.075		8
	M7 (beta)	−2,649.889	95.034	
	M8 (beta&ω)	−2,602.372		8
*rpoA*	M0 (one ratio)	ω = 0.425		
	M1 (neutral)	−1960.904	18.516	
	M2 (selection)	−1951.646		9
	M7 (beta)	−1961.168	19.006	
	M8 (beta&ω)	−1951.665		9
*rpoB*	M0 (one ratio)	ω = 0.170		
	M1 (neutral)	−6,023.244	25.005	
	M2 (selection)	−6,010.741		9
	M7 (beta)	−6,023.711	25.873	
	M8 (beta&ω)	−6,010.775,053		9
*rpoc1*	M0 (one ratio)	ω = 0.263		
	M1 (neutral)	−4,017.309	50.238	
	M2 (selection)	−3,992.190		11
	M7 (beta)	−4,018.563	51.186	
	M8 (beta&ω)	−3,992.970		11
*rpoc2*	M0 (one ratio)	ω = 0.528		
	M1 (neutral)	−10233.557	465.104	
	M2 (selection)	−10001.005		45
	M7 (beta)	−10233.873	461.100	
	M8 (beta&ω)	−10003.323		48
*rps8*	M0 (one ratio)	ω = 0.524		
	M1 (neutral)	−856.327	14.975	
	M2 (selection)	−848.840		4
	M7 (beta)	−854.884	12.935	
	M8 (beta&ω)	−848.417		5
*rps11*	M0 (one ratio)	ω = 0.273		
	M1 (neutral)	−746.510	7.472	
	M2 (selection)	−742.774		1
	M7 (beta)	−746.512	7.466	
	M8 (beta&ω)	−742.779		1
*rps19*	M0 (one ratio)	ω = 0.320		
	M1 (neutral)	−603.312	16.924	
	M2 (selection)	−594.850		3
	M7 (beta)	−603.357	17.004	
	M8 (betaω)	−594.855		3
*ycf1*	M0 (one ratio)	ω = 0.632		
	M1 (neutral)	−14774.834	157.711	
	M2 (selection)	−14695.978		42
	M7 (beta)	−14774.915	156.596	
	M8 (beta&ω)	−14696.617		61
*ycf2*	M0 (one ratio)	ω = 0.840		
	M1 (neutral)	−10754.288	44.430	
	M2 (selection)	−10732.073		81
	M7 (beta)	−10754.623	45.095	
	M8 (beta&ω)	−10732.076		81
*ycf3*	M0 (one ratio)	ω = 0.161		
	M1 (neutral)	−899.336	16.519	
	M2 (selection)	−891.077		1
	M7 (beta)	−900.724	19.279	
	M8 (beta&ω)	−891.084		1

The positive selection of cp genes has been widely studied in angiosperms and demonstrated at the protein level (Li et al., 2020). In this study, *psbK* and *psbI*, the subunits of the cp photosynthetic system (Li et al., 2019), were positively selected in *Acer*. To our knowledge, the positive selection of *psbK* and *psbI* was not common in angiosperms. The high ω implies a unique attribute of *Acer* to adapt to different light environments. Most of the 20 genes with codons positively selected detetced under the BEB algorithm had one or two positively selected codons, but the *ycf1*, *ycf2*, and *rpoC2* genes contained more than 40 sites under selection. Although we don’t have enough evidence to make definite inferences, past researches, for example, have indicated that *ycf1* is exceptionally divergent across land plants (Dong et al., 2015; Mower et al., 2019) and *rpoC2* had the most positive selective sites among the cp genes in *Siraitia* species (Shi et al., 2019). They indicated that *rpoC2* had a higher evolutionary rate in several species. These genes that undergo positive selection might result from adaptation to specific ecological niches.

### 3.5 Phylogenetic Analysis

**FIGURE 4:**
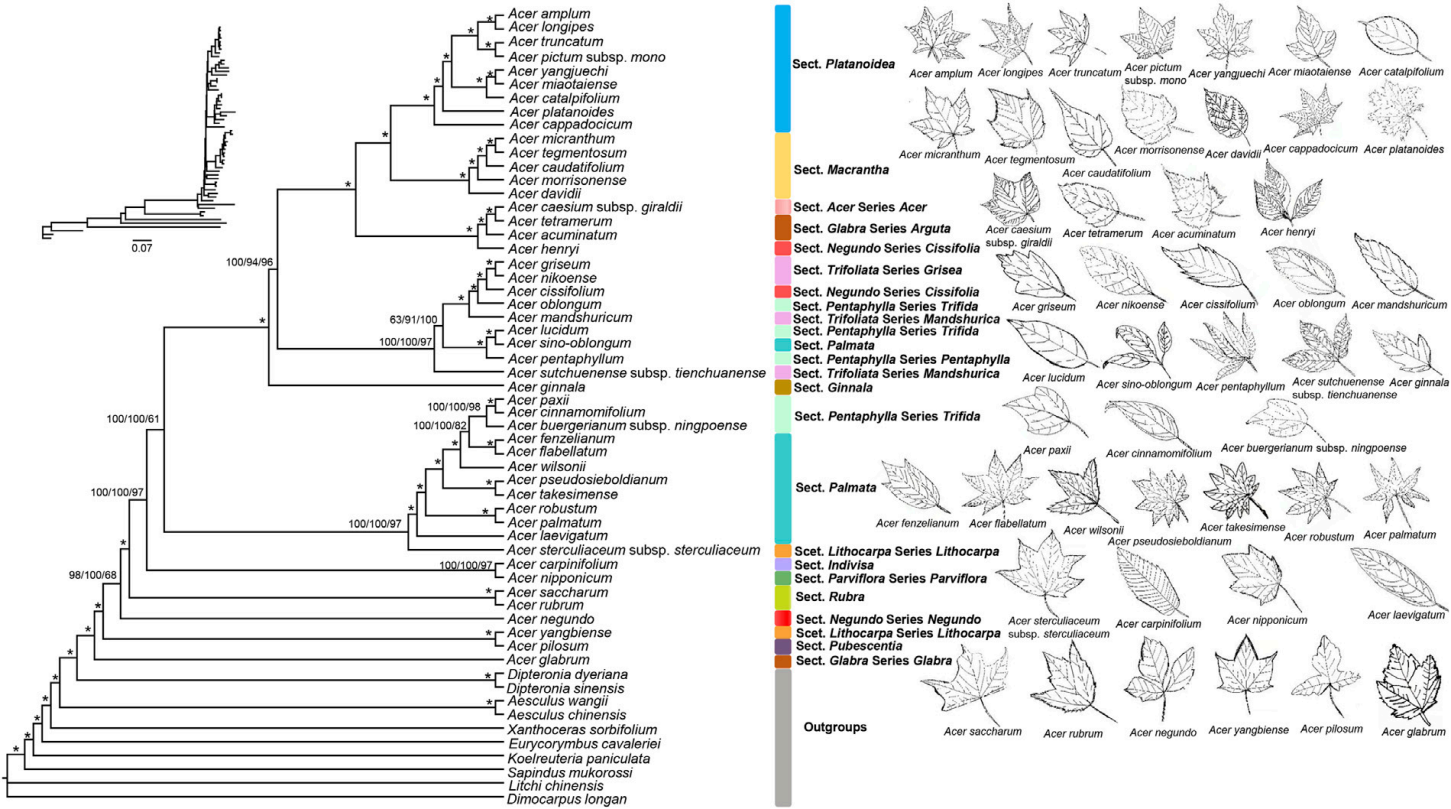
Phylogenetic tree of 48 *Acer* species inferred by Bayesian Inference (BI), Maximum Likelihood (ML) and Maximum Parsimony (MP) methods, based on the whole cp genome sequences. The numbers above the branches are the posterior probabilities of BI and bootstrap values of ML and MP. Asterisks represent nodes with maximal support values in all methods. Each Section was marked in the same colour.

Most nodes of the reconstructed phylogenomic tree had 100% bootstrap support values, indicating a suitable evolutionary placement for *Acer* species (Figure 4). The results showed that *Acer* and *Dipteronia* are monophyly, which is consistent with previous studies (Renner et al., 2008; Gao et al., 2020; Wang et al., 2020; Areces-Berazain et al., 2021)*. Acer pictum* subsp. *mono* is traditionally considered sister to *A*. *truncatum* but not to *A*. *yangjuechi* (van Gelderen et al., 1994; Xu et al., 2008). However, Yu et al. (2020) proposed *A*. *pictum* subsp. *mono* and *A*. *yangjuechi* as sister species according to the “local varieties.” The leaves of *A. pictum* subsp. *mono* and *A*. *truncatum* has 5-lobed and glabrous abaxially, while *A*. *yangjuechi* (synonym for *A*. *miaotaiense* in *Maples of the World* and *Flora of China*) is 3-lobed, undulate margin and obtuse lobes. In addition, our study showed that each branch within the *Platanoidea* section had high support, which is consistent with morphological classification (van Gelderen et al., 1994; Xu et al., 2008). Our results strongly support that section *Platanoidea* and section *Macrantha* are sister sections (Figure 4), similar to previous studies (Renner et al., 2008; Areces-Berazain et al., 2020; Wang et al., 2020). The morphological characteristics of the two sections are similar, such as simple leaves with 3- or 5-lobed or unlobed (Xu et al., 2008). However, this result is still inconsistent with some studies, such as Li et al. (2019) and Areces-Berazain et al. (2021), which may be due to different marker selection and single model in the phylogenetic analysis.

In *Maples of the World*, section *Glabra* comprises species from the *Glabra* and *Arguta* series (van Gelderen et al., 1994). However, many studies, including the present one, have shown a certain genetic distance between these two series (Li et al., 2019; Areces-Berazain et al., 2020; Gao et al., 2020; Areces-Berazain et al., 2021). Series *Glabra* is monotypic, containing only *A*. *glabrum* and its subspecies. They are mainly shrubs with 5-merous and 8-stamens flowers distributed in North America, unlike Series *Arguta*, with 4-merous and 4-6 stamens distributed in East Asia (van Gelderen et al., 1994). Therefore, dividing the two series into two sections is more appropriate, as de Jong (2004) proposed. Species of sections *Trifoliata* and *Pentaphylla* were mixed (Figure 4), suggesting their sister relationship (Li et al., 2019; Gao et al., 2020; Wang et al., 2020; Areces-Berazain et al., 2021). These two sections have compound leaves, distinguishing them from most other sections in *Acer* (Xu et al., 2008). The Section *Palmata* was not monophyletic as it lacked *A*. *sino*-*oblongum*, which is consistent with previous studies (Gao et al., 2017; Wang et al., 2020). Although many studies have placed *A*. *sino*-*oblongum* in Section *Palmata* (van Gelderen et al., 1994; Xu et al., 2008), the taxonomic status of this species must be revisited. *Acer yangbiense*, a rare and critically endangered species, is herein shown to be genetically distant from the other species in Section *Lithocarpa*, as in previous studies (Li et al., 2019; Areces-Berazain et al., 2021). This species has pale white to pale gray leaf blade abaxially, entire leaf margin, and slender fruiting pedicels, which are pretty different from other species in the Section *Lithocarpa* (van Gelderen et al., 1994; Xu et al., 2008). Determining the systematic position of *A*. *yangbiense* is of great significance to conserving this rare and endangered species.

## 4 Conclusion

This study compared 48 whole cp genome sequences of *Acer*, which exhibited a typical quadripartite structure and genomic content. The comparative study allowed us to identify hotspot loci and several transferable polymorphic SSR applied as DNA barcodes for species identification and phylogenetic inference. Moreover, the complete plastome data allowed us to obtain the highest phylogenetic resolution to date for the 48 *Acer* species, showing that the cp phylogenomic approach could be employed to tackle the intractable phylogenic problems in *Acer*. The comparative genomic information constitutes a valuable resource in advancing our understanding of plastid evolution and molecular breeding application for the agro-horticulture in *Acer* species.

## Data Availability

The original contributions presented in the study are included in the article/Supplementary Material, further inquiries can be directed to the corresponding author.
