# Supplementary material for: Insights Into Comparative Analyses and Phylogenomic Implications of Acer (Sapindaceae) Inferred From Complete Chloroplast Genomes
Source: Front Genet. 2022 Jan 3;12:791628. doi: 10.3389/fgene.2021.791628 (PMC8762318; doi:10.3389/fgene.2021.791628)
Supplement: Supplementary file 1 [file DataSheet1.docx]

Supplementary Material

**
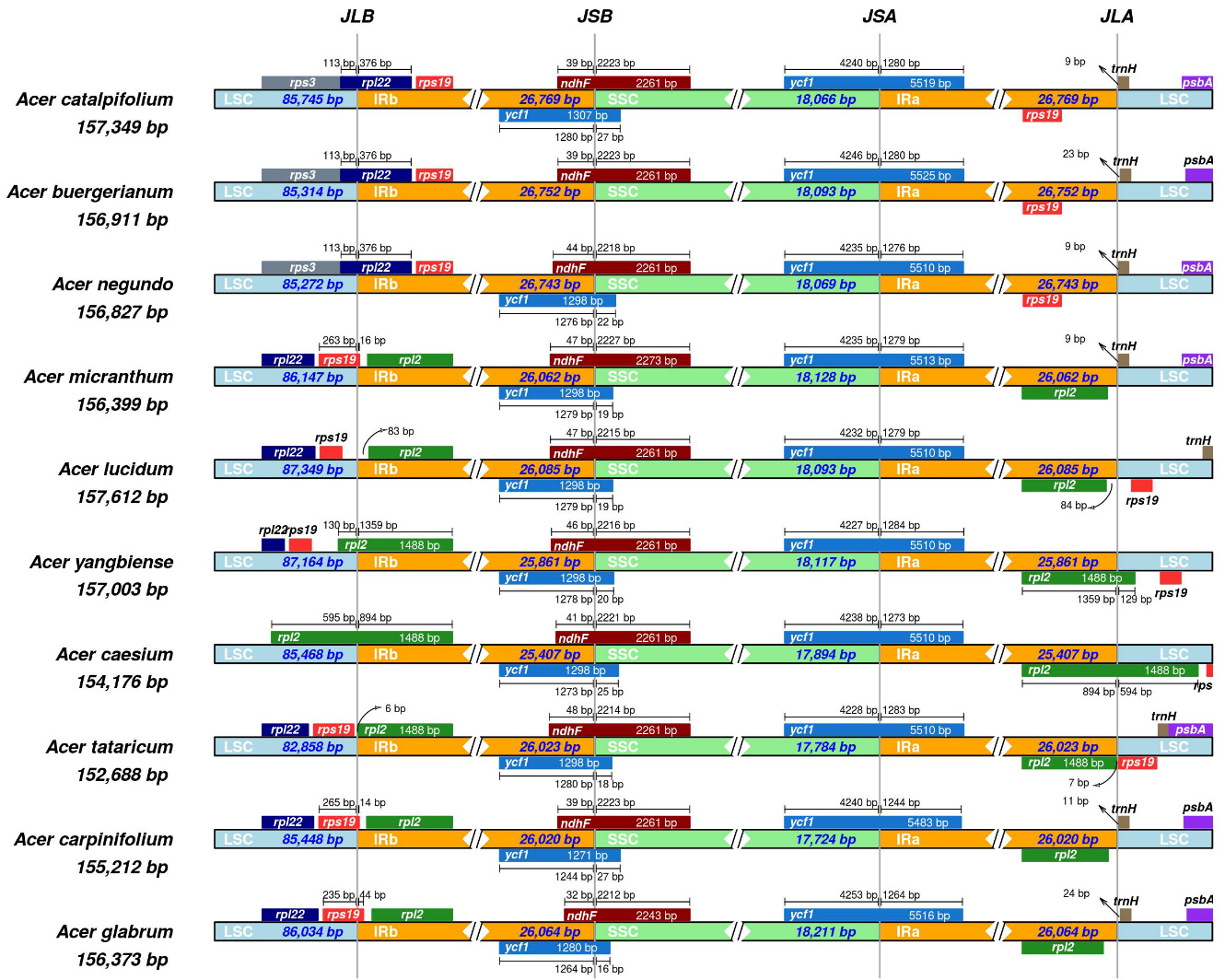
**

**FIGURE S1** Comparison of the junction sites of the LSC, IRs and SSC regions among 10 *Acer* species chloroplast genomes.

**TABLE S1** The collecting information of *Acer* leaf materials.

| **Species** | **Section** | **Source** | **Native geographic region** |
| --- | --- | --- | --- |
| *Acer palmatum* Thunberg ex Murray | *Palmata* | Institute of Botany, Chinese Academy of Sciences, Beijing, China | Widespread in Japan, Korea, eastern China. at 200-1200 m. |
| *Acer flabellatum* (Rehder) Murray | *Palmata* | Taian Arboretum, Taian, China | Hubei and Sichuan Provinces, China, at 1800-2600 m. Also occurring in Yunnan Province, northern Laos, and Vietnam. |
| *Acer sino*-*oblongum* Metc. | *Palmata* | Qingxiushan, Nanning, China | Guangdong and Guangxi Provinces, China, at 0-100 m. |
| *Acer laevigatum* Wallich | *Palmata* | Shanghai Botanical Garden, Shanghai, China | Yunnan, Shaanxi, and Hubei Provinces, China, at 1200-1800 m; Nepal. |
| *Acer wilsonii* (Rehder) de Jong, comb. & stat. nov. | *Palmata* | Qingxiushan, Nanning, China | Hubei, Yunnan, Zhejiang, Guangdong, Guangxi Provinces, China, at 1200-1800 m. |

**TABLE S2** Outgroups of phylogenetic tree in this study.

| **Species** | **Accession No.** |
| --- | --- |
| *Dimocarpus longan* | MW067098 |
| *Litchi chinensis* | MW067100 |
| *Sapindus mukorossi* | NC025554 |
| *Koelreuteria paniculata* | NC037176 |
| *Eurycorymbus cavaleriei* | MK552106 |
| *Xanthoceras sorbifolium* | NC037448 |
| *Aesculus wangii* | NC035955 |
| *Aesculus chinensis* | MK648235 |
| *Dipteronia* *sinensis* | NC031899 |
| *Dipteronia* *dyeriana* | KT878501 |
